# Supplementary material for: Nucleoli-localized KANSL2 as an epigenetic regulator of ribosome biogenesis in glioblastoma cells
Source: Commun Biol. 2026 Mar 5;9:535. doi: 10.1038/s42003-026-09808-3 (PMC13086876; doi:10.1038/s42003-026-09808-3)

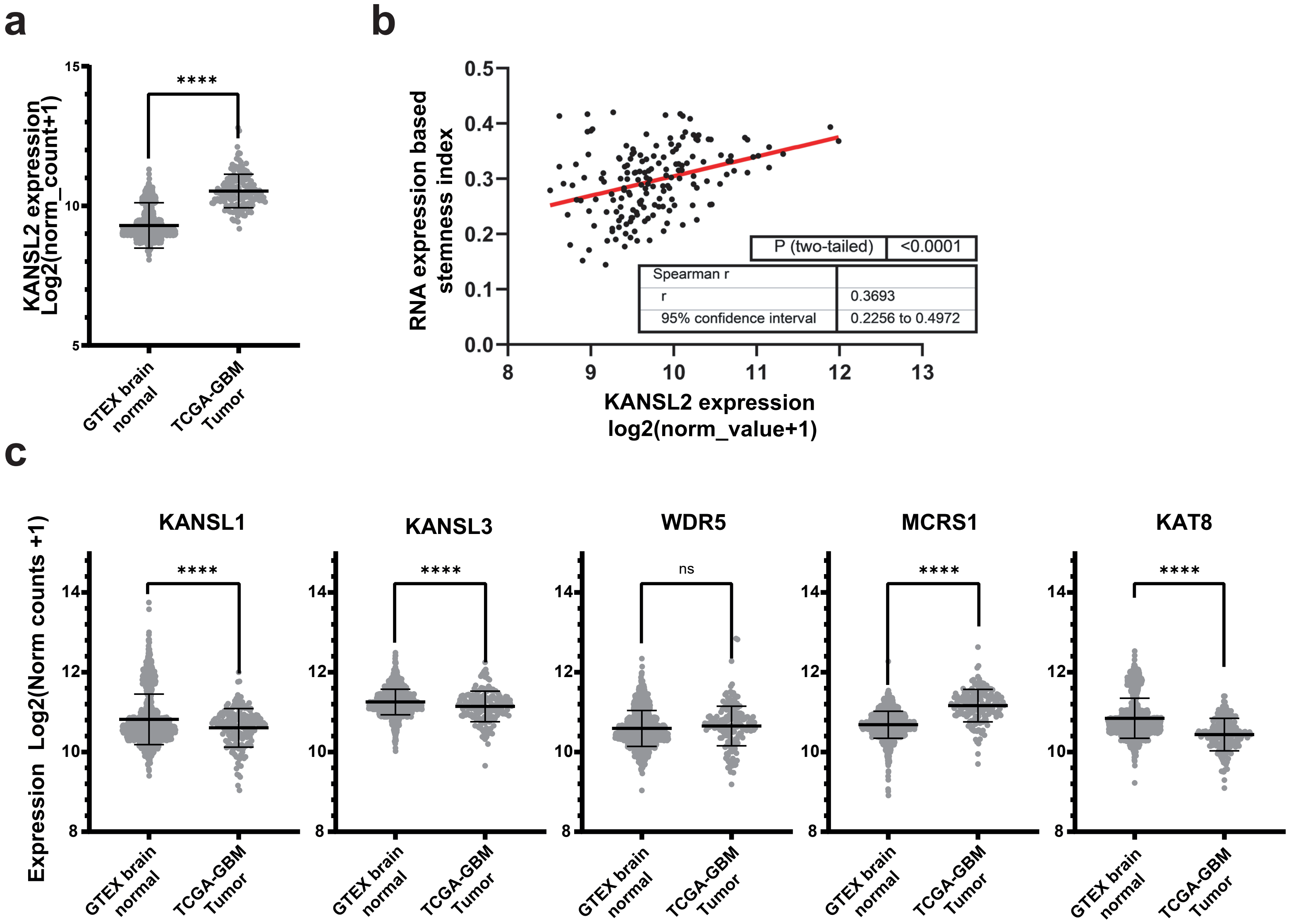

Supplementary Figure 1

a) KANSL2 expression of GTEX brain normal tissue samples vs TCGA-GBM tumor samples. (*t-test* was performed; **** *p* <0.001; data are represented as mean ± SD) **b)** Spearman's correlation and linear regression analysis of KANSL2 expression versus stemness index score (*t-test* was performed; n = 166 patient samples; **** *p* ≤0.0001). **c)**Transcriptomic analysis of NSL complex members (KANSL1, KANSL3, WDR5, MCRS1 and KAT8) in TCGA-GBM cancer. A column scatter plot showing the normalized (log2 (normcount +1)) mRNA expression of NSL complex members in GTEX brain normal tissue (n = 1136 patient samples) versus TCGA-GBM primary tumor (n = 166 patient samples) (*t-test* was performed, **** *p* ≤0.0001; data are represented as mean ± SD).

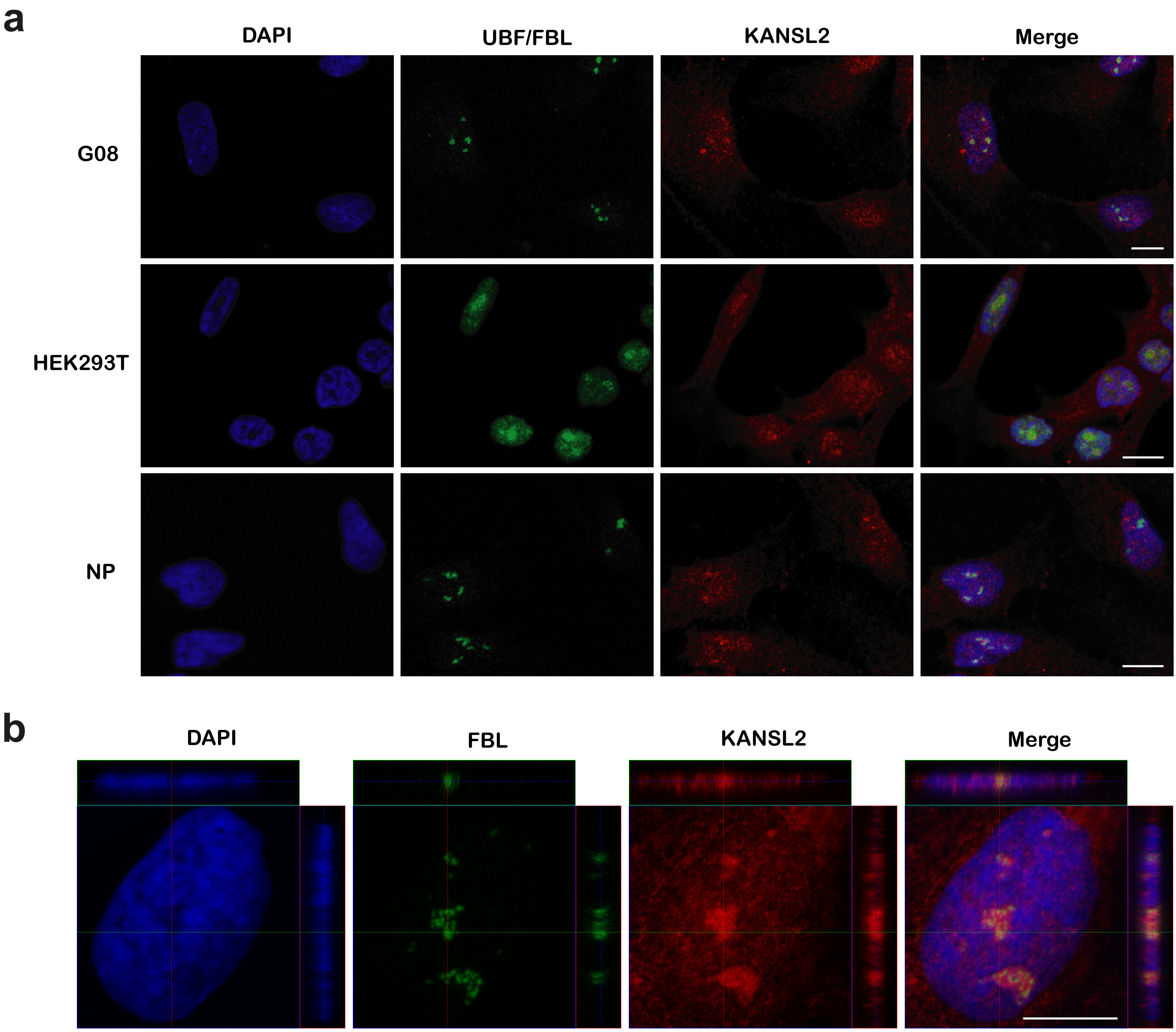

Supplementary Figure 2

a) Immunofluorescence detection of endogenous KANSL2 and nucleolar markers (UBF or FBL) in GBM patient-derived cancer stem cells enriched G08 line, HEK293T (HEK) cells, and neural progenitor cells (NP). DAPI was used to stain the nuclei (scale bar, 10μm). **b)** Immunofluorescence detection of endogenous KANSL2 and FBL in U251 GBM cells. DAPI was used to stain the nuclei Z-Stack of the orthogonal image (scale bar, 10μm).

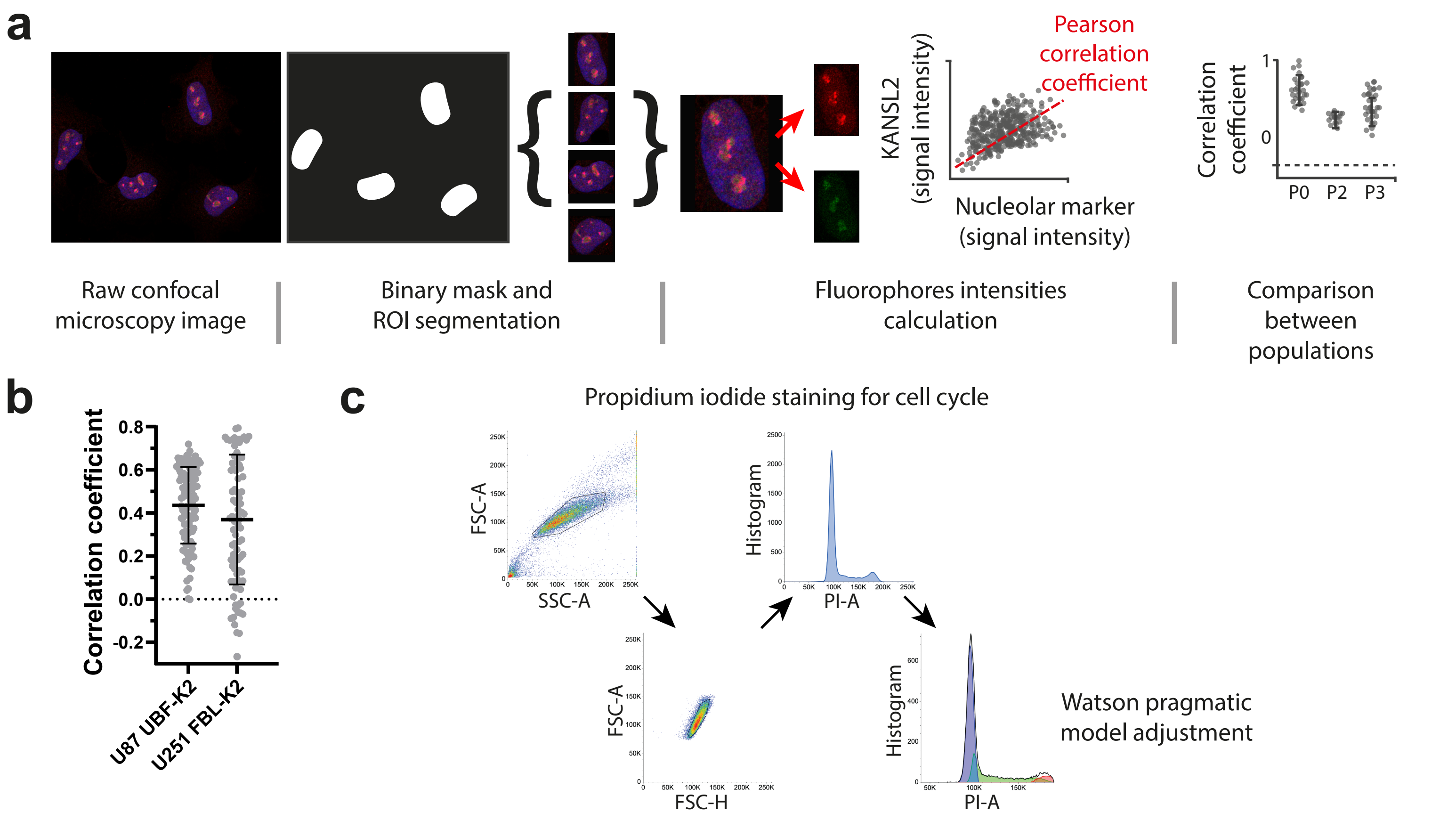

Supplementary Figure 3

a) Flowchart describing the quantification of KANSL2 nucleolar localization, starting from raw multi-channel inmunofluorescence images (KANSL2, UBF, DAPI and bright field), applying DAPI-based binary mask, isolating regions of interest (ROIs) and obtaining Pearson correlation coefficient values. **b)** Correlation coefficient (i.e. Pearson's correlation coefficient of pixels intensity) (n =108 nuclei for U87 UBF-K2; n=86 nuclei for U251 FBL-K2). **c)** Gating strategy for cell cycle analysis using PI. Debris and doublets were excluded using FSC-A vs. FSC-H and SSC-A gating. DNA content was analyzed using linear PI fluorescence, and cell cycle phases (G0/G1, S, G2/M) were defined by applying the Watson Pragmatic model in Floreada.io.

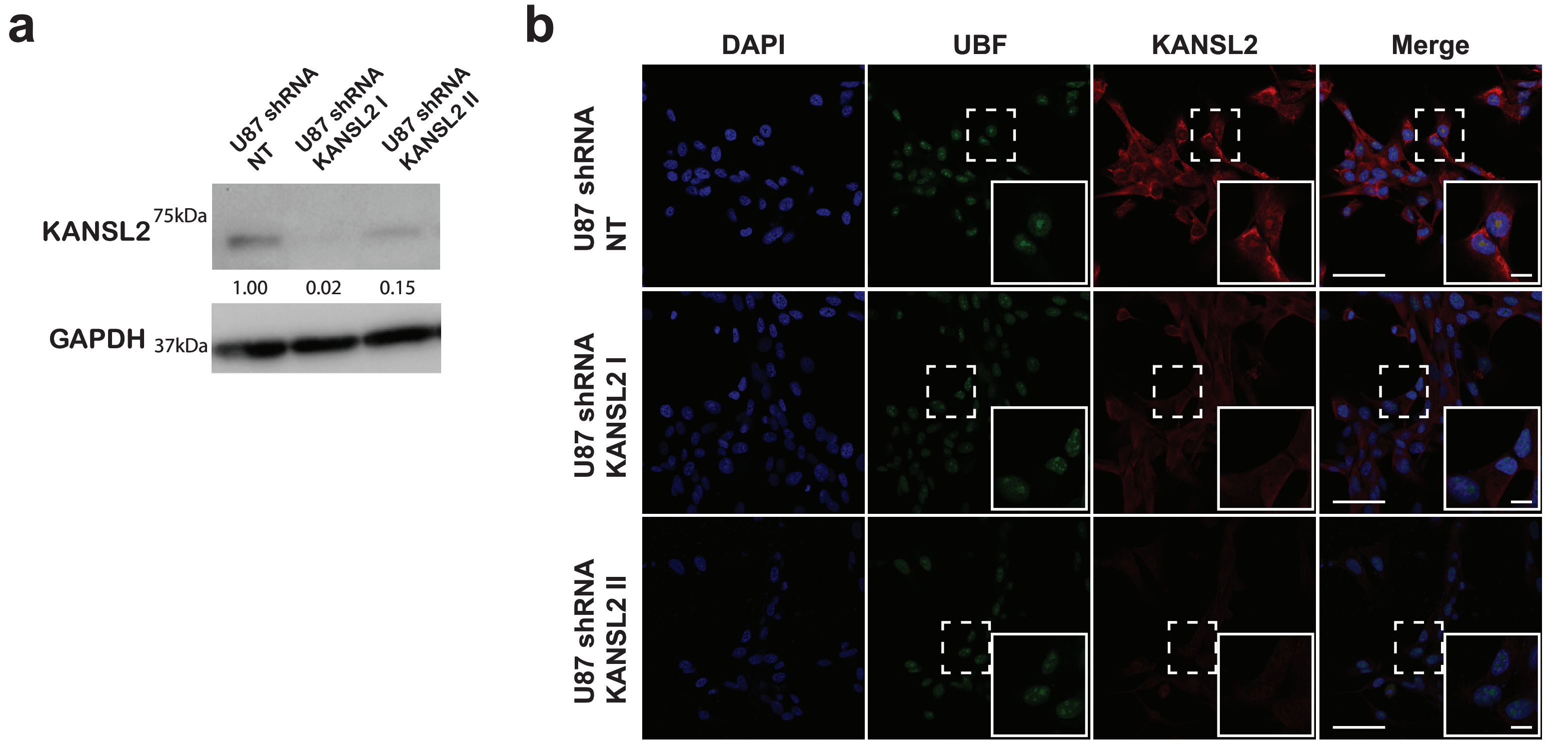

Supplementary Figure 4

a) Western blot analysis showing KANSL2 protein levels in U87 stably infected with 2 different KANSL2 shRNAs (I and II). **b)** Immunofluorescence detection of endogenous KANSL2 and nucleolar UBF in U87 cells KANSL2-depleted cells indicating a general reduction of KANSL2 expression signal. (scale bars, 50μm and 10 μm for insets).

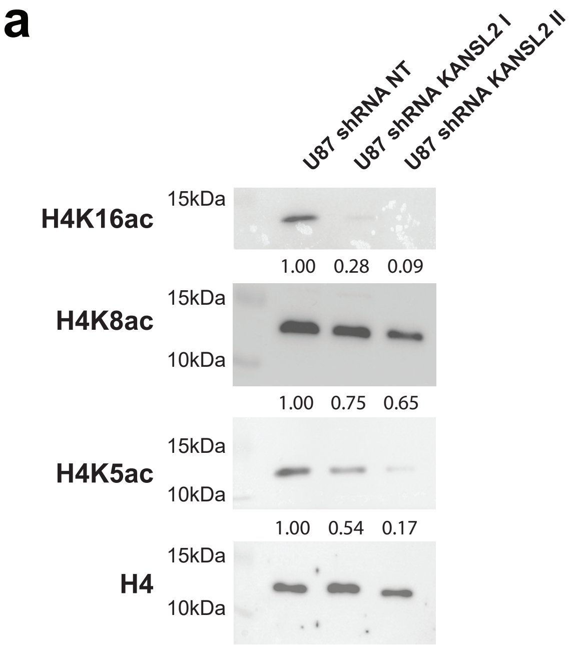

Supplementary Figure 5

a) Western blotting analysis of H4K16ac, H4K5ac, H4K8ac and H4 levels after KANSL2 KDs in U87 cells.

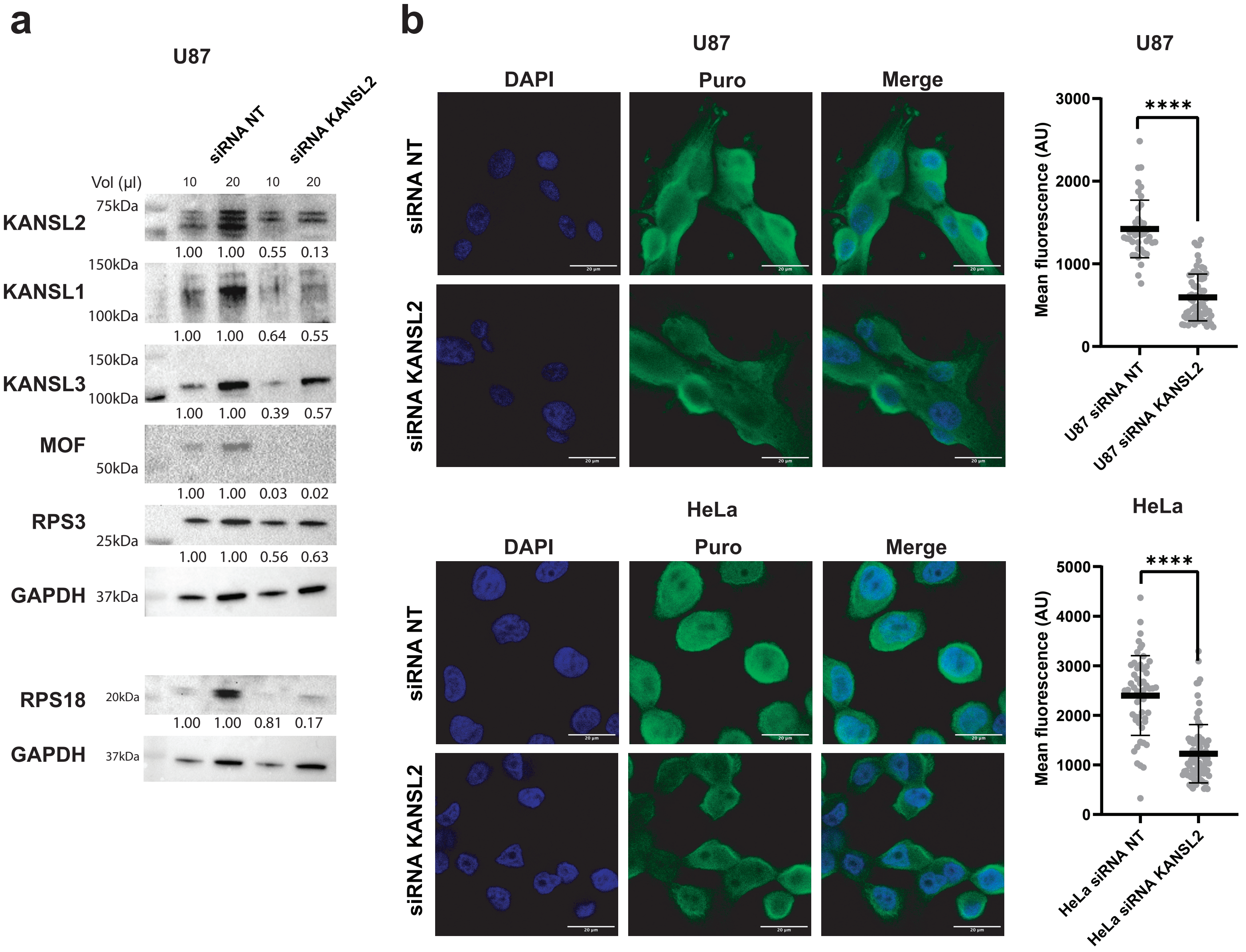

Supplementary Figure 6

a) Western blotting analysis of KANSL2, KANSL1, KANSL3, MOF, RPS3, RPS18 and GAPDH levels in siRNA NT and siRNA KANSL2 U87 cells. **b)** Representative confocal images and quantification of puromycin signals in U87 (n = 43 cells for siRNA NT y n= 70 cells for siRNA KANSL2) and HeLa (n = 54 cells for siRNA NT y n= 83 cells for siRNA KANSL2) cells after 48 h from siRNA application and incubated with puromycin antibody. DAPI was used for nuclei staining (*t-test* was performed; **** $p \leq 0.0001$; scale bar, 20µm).

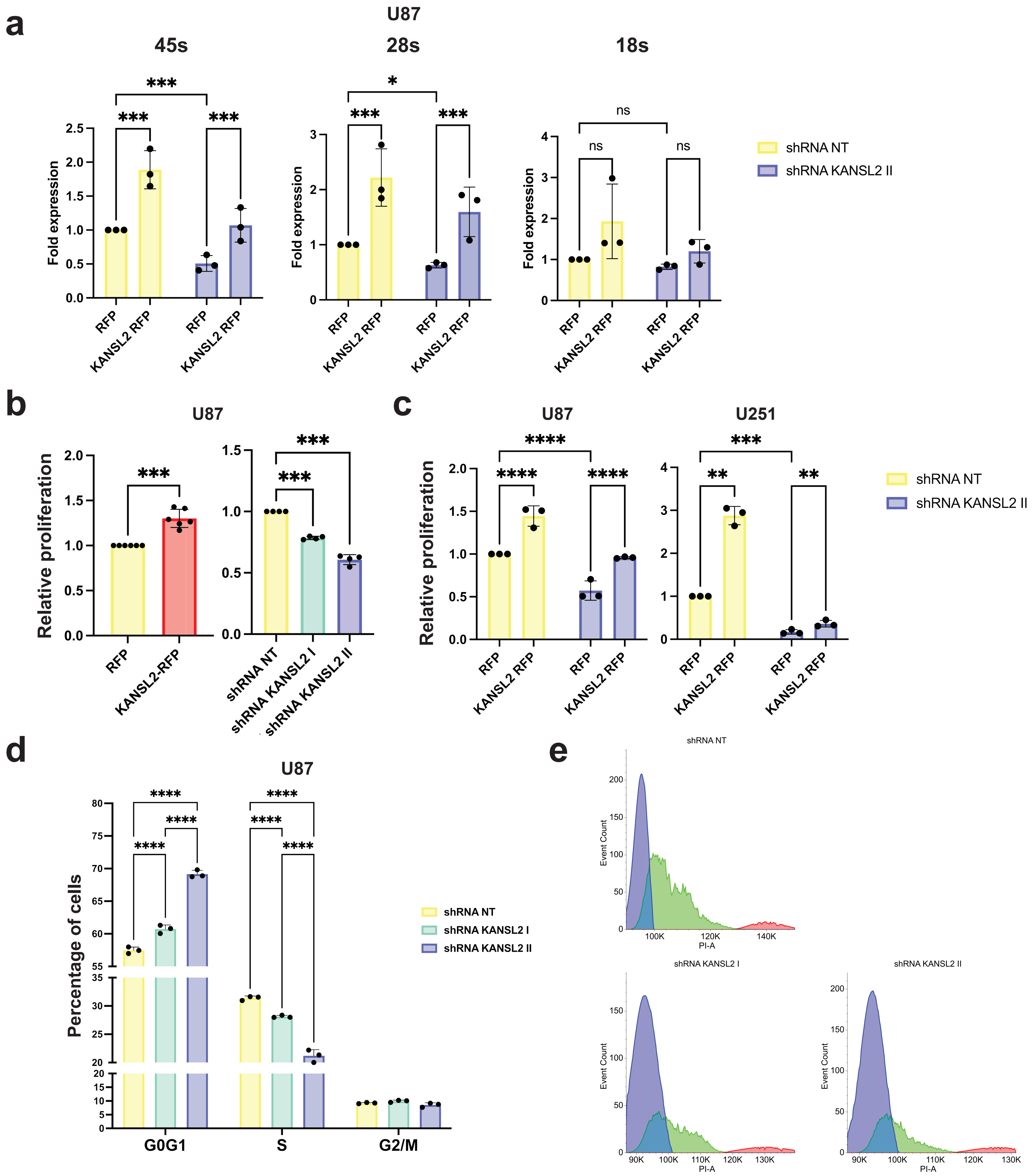

Supplementary Figure 7

a) RT–qPCR analysis of 45S pre-rRNA, 28S, and 18S rRNA levels in U87 cells expressing the indicated constructs: non-targeting control (NT) + RFP, NT + KANSL2–RFP, shKANSL2-II + RFP, and shKANSL2-II + KANSL2–RFP (two-way ANOVA followed by Fisher´s least significant difference (LSD) post hoc test (uncorrected) was performed; n=3; * $p \leq 0.05$, *** $p \leq 0.001$; data are shown as mean ± SD). **b)** Crystal violet proliferation assay in U87 cells with stable KANSL2 knockdown or overexpression (one-way ANOVA followed by Dunnett's test was performed; n=3; *** $p \leq 0.001$; data are shown as mean ± SD) **c)** Crystal violet proliferation assay in U87 and U251 cells expressing the indicated constructs: NT + RFP, NT + KANSL2–RFP, shKANSL2-II + RFP, and shKANSL2-II + KANSL2–RFP (two-way ANOVA followed by Fisher's LSD post hoc test (uncorrected) was performed; n=3; ** $p \leq 0.01$, *** $p \leq 0.001$, **** $p \leq 0.0001$; data are shown as mean ± SD). **d)** Cell-cycle analysis of U87 cells after KANSL2 depletion, determined by propidium iodide staining and flow cytometry. The percentage of cells in G0/G1, S, and G2/M phases is shown (one-way ANOVA followed by Dunnett's test was performed; n=3; **** $p \leq 0.0001$; data are shown as mean ± SD). **e)** Representative histograms from cell-cycle analysis.

Supplementary Table S1 _ Antibodies

Antibody Target	Catalog	Research Resource Identifiers (RRID)	Supplier	Application
KANSL2	HPA038497	RRID:AB_10674685	Sigma-Aldrich	WB (1:500) and IF (1:250)
KANSL1	STJ112653	RRID: AB_3665870	St John´s laboratories	WB (1:500)
KANSL3	STJ110533	RRID: AB_3665871	St John´s laboratories	WB (1:500)
KAT8/MOF	Ab200660	RRID:AB_2891127	Abcam	WB (1:1000)
FBL	sc166000	RRID:AB_2105803	Santa Cruz Biotechnology	IF (1:1000)
UBF	sc13125	RRID:AB_671403	Santa Cruz Biotechnology	IF (1:250)
PUROMYCIN	MABE343	RRID:AB_2566826	Merck	WB (1:25000) and IF (1:1000)
Beta-Actin	sc-47778	RRID:AB_626632	Santa Cruz Biotechnology	WB (1:1000)
RPS3	9538	RRID:AB_10622028	Cell signaling technologies	WB (1:1000)
RPS18	Ab91293	RRID:AB_2050267	Abcam	WB (1:500)
GAPDH	ab8245	RRID:AB_2107448	Abcam	WB (1:3000)
H4	ab10158	RRID:AB_296888	Abcam	WB (1:1000)
H4K5ac	8647	RRID:AB_11217428	Cell signaling technologies	WB (1:1000) and ChIP (1 µg: 200000 cells)
H4K8ac	ab45166	RRID:AB_732937	Abcam	WB (1:10000) and ChIP (1 µg: 200000 cells)

H4K16ac	13534	RRID:AB_2687581	Cell signaling technologies	WB (1:1000)
Goat Anti-Mouse IgG-HRP	1706516	RRID:AB_2921252	BIORAD	WB (1:3000)
Goat Anti-Rabbit IgG-HRP	1721019	RRID:AB_11125143	BIORAD	WB (1:3000)
Goat anti-Mouse IgG (H+L) Highly Cross-Adsorbed Secondary Antibody, Alexa Fluor™ Plus 488	A32723	RRID:AB_2633275	Thermo Fisher	IF (1:1000)
Goat anti-Rabbit IgG (H+L) Highly Cross-Adsorbed Secondary Antibody, Alexa Fluor™ Plus 555	A32732	RRID:AB_2633281	Thermo Fisher	IF (1:1000)

Supplementary Table S2 _ Primers qRT-PCR

Primer	Sequence (5’-3’)
Human KANSL2 Forward	CACAGGCAACCCAGACTACC
Human KANSL2 Reverse	CCCTGAGTGGGAAGAAACCC
Human KAT8 Forward	CTTGTCTACCCACTCGTCCAG
Human KAT8 Reverse	CGGAGAAACGTACCTGTGCC
Human POLR1E Forward	ATCCTTGGCCGGCACTTTGAG
Human POLR1E Reverse	TCCACCTGCGGCTTGATGTTC
Human UBF Forward	ACCAGCCACCTCCGAACAG
Human UBF Reverse	AGGCAGGCTCTCGAGGAAAC
Human 45s Forward	GAACGGTGGTGTGTCGTTCC
Human 45s Reverse	GCGTCTCGTCTCGTCTCACT
Human 28s Forward	AGAGGTAAACGGGTGGGGTC
Human 28s Reverse	GGGGTCGGGAGGAACGG
Human 18s Forward	GTAACCCGTTGAACCCCATT
Human 18s Reverse	CCATCCAATCGGTAGTAGCG
Human Beta Actin Forward	AGAGCTACGAGCTGCCTGAC
Human Beta Actin Reverse	AGCACTGTGTTGGCGTACA

Human GAPDH Forward	GTGAAGGTCGGAGTCAACGG
Human GAPDH Reverse	TGATGACAAGCTTCCCGTTCTC

Supplementary Table S3 _ Primers qPCR CHIP assay

Primer	Sequence (5'-3')
rRNA H4 Forward	CGACGACCCATTCGAACGTCT
rRNA H4 Reverse	CTCTCCGGAATCGAACCCTGA
rRNA H8 Forward	AGTCGGGTTGCTTGGGAATGC
rRNA H8 Reverse	CCCTTACGGTACTTGTTGACT
rRNA H42.9 Forward	CCCGGGGGAGGTATATCTTT
rRNA H42.9 Reverse	CCAACCTCTCCGACGACA
rRNA UCE Forward	CTCCCGCTCTGGAGACAC
rRNA UCE Reverse	GGACACCTGTCCCCAAAAAC
Human NAGPA TSS Forward	GTCGCCATATTGGACCGGG
Human NAGPA TSS Reverse	GCGCAGGTAATTCAGTCCGA
Human GENE DESERT (OPEN) Forward	GGGTGCAAGGAGTGGCTTAT
Human GENE DESERT (OPEN) REVERSE	GAATGGCCAATTGAGCTGCC

Uncropped Blots

Figure 2

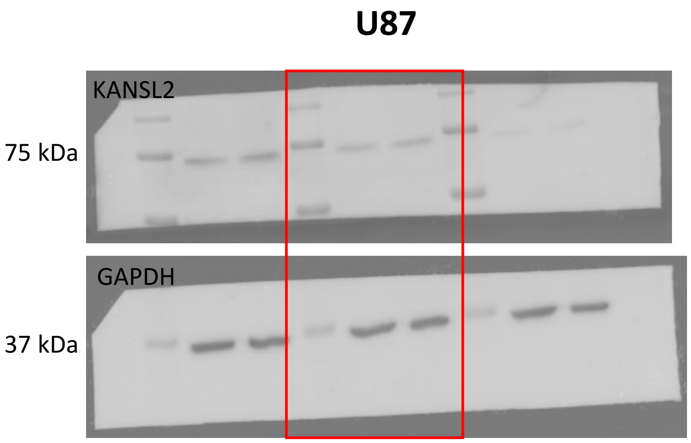

Figure 3

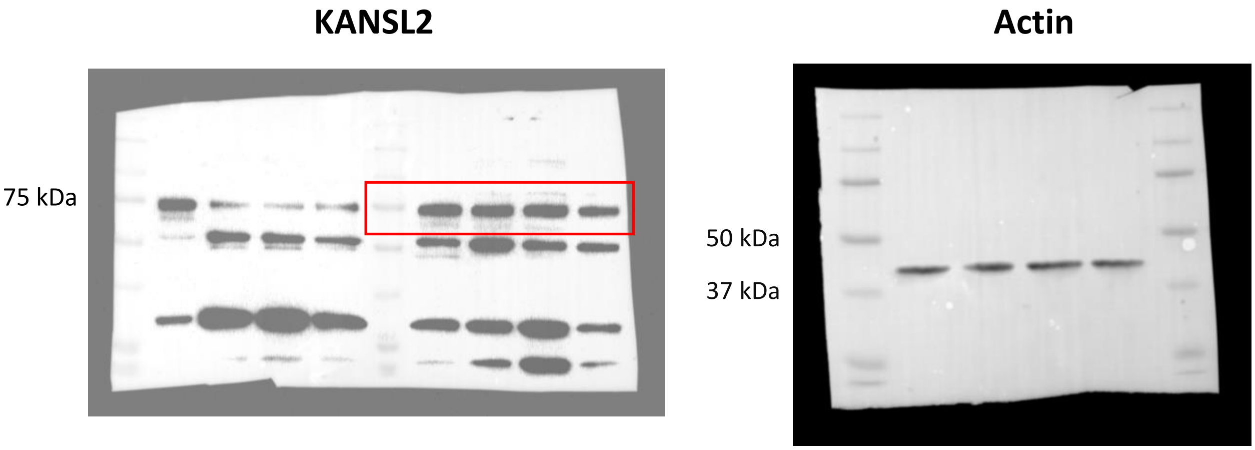

Figure 5

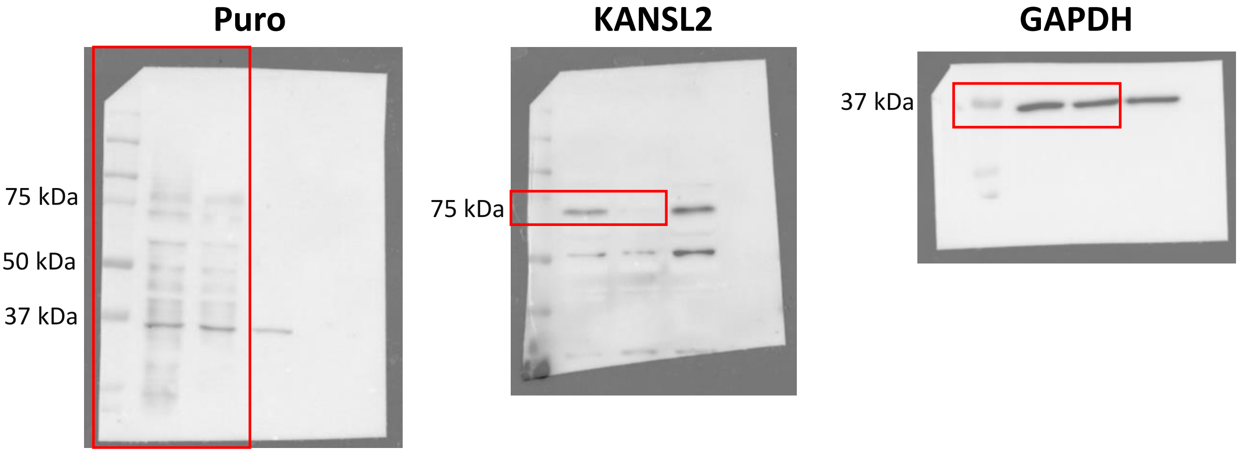

Supp Figure 4

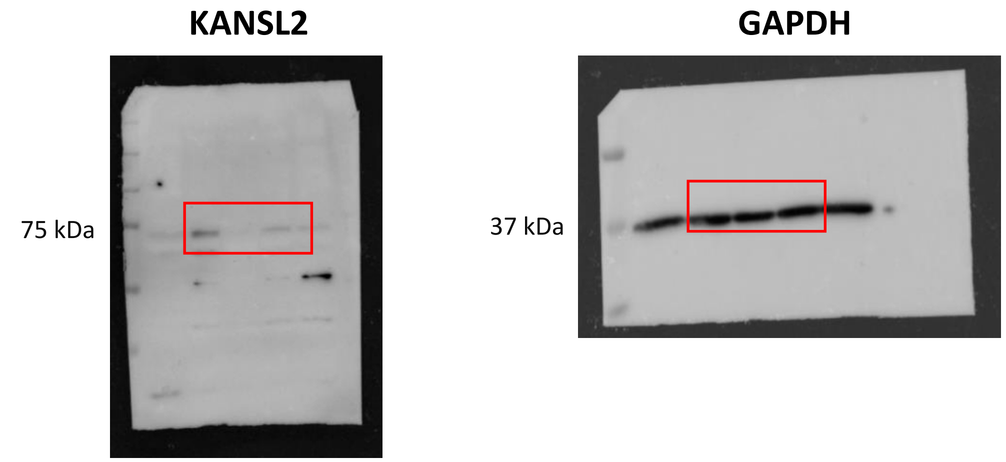

Supp Figure 5

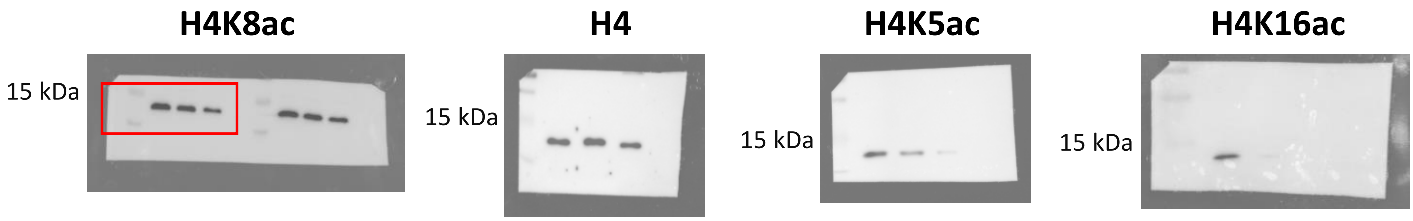

Supp Figure 6

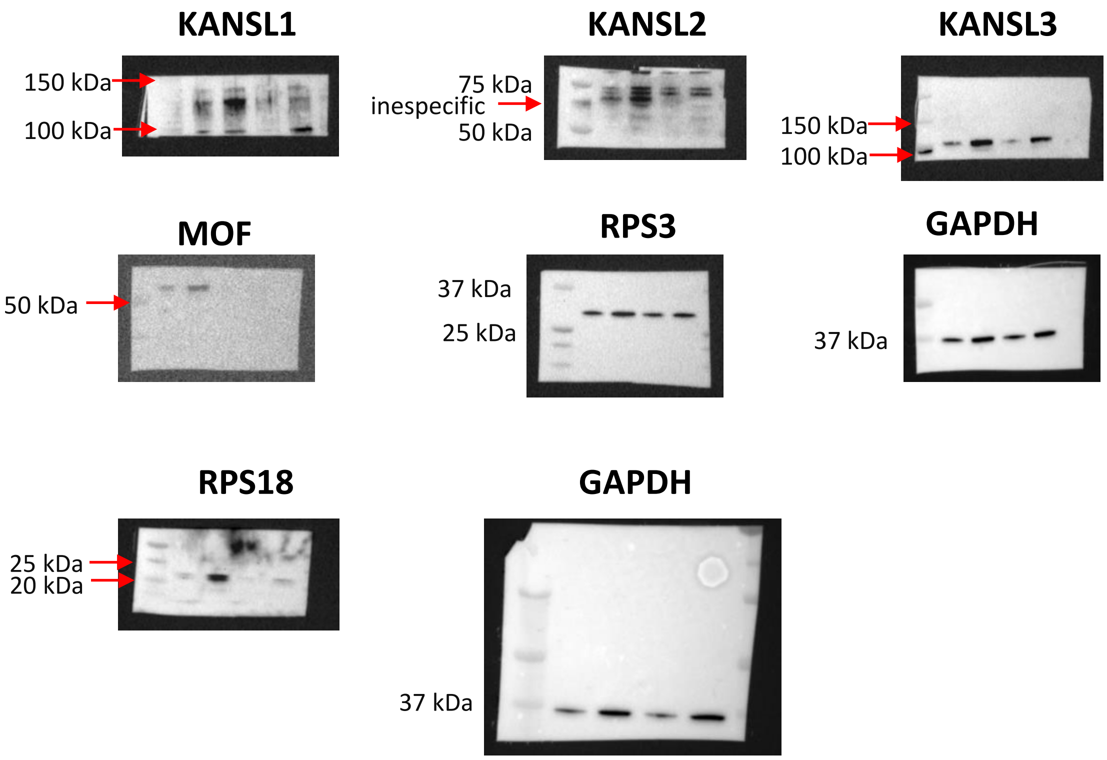

Supplement: Supplementary file 1 — Supplementary info [file 42003_2026_9808_MOESM1_ESM.pdf]
