# Supplementary material for: Nucleoli-localized KANSL2 as an epigenetic regulator of ribosome biogenesis in glioblastoma cells
Source: Commun Biol. 2026 Mar 5;9:535. doi: 10.1038/s42003-026-09808-3 (PMC13086876; doi:10.1038/s42003-026-09808-3)
Supplement: Supplementary file 5 — Description of Additional Supplementary files [file 42003_2026_9808_MOESM5_ESM.pdf]

Description of Additional Supplementary Files

File Name: Supplementary Data 1

Description: Data set Fig 2 to 6

File Name: Supplementary Data 2

Description: TCGA data

File Name: Supplementary Data 3

Description: Data for calculation of Pearsons coefficients correlations
