# Supplementary material for: Nucleoli-localized KANSL2 as an epigenetic regulator of ribosome biogenesis in glioblastoma cells
Source: Commun Biol. 2026 Mar 5;9:535. doi: 10.1038/s42003-026-09808-3 (PMC13086876; doi:10.1038/s42003-026-09808-3)
Supplement: Supplementary file 6 — Reporting summary [file 42003_2026_9808_MOESM6_ESM.pdf]

nature portfolio

Corresponding author(s): Perez-Castro, Carolina

Last updated by author(s): Jan 26, 2026

# Reporting Summary

Nature Portfolio wishes to improve the reproducibility of the work that we publish. This form provides structure for consistency and transparency in reporting. For further information on Nature Portfolio policies, see our Editorial Policies and the Editorial Policy Checklist.

## Statistics

For all statistical analyses, confirm that the following items are present in the figure legend, table legend, main text, or Methods section.

| n/a | Confirmed | |
|---|---|---|
| ☐ | ☒ | The exact sample size (*n*) for each experimental group/condition, given as a discrete number and unit of measurement |
| ☐ | ☒ | A statement on whether measurements were taken from distinct samples or whether the same sample was measured repeatedly |
| ☐ | ☒ | The statistical test(s) used AND whether they are one- or two-sided<br>*Only common tests should be described solely by name; describe more complex techniques in the Methods section.* |
| ☐ | ☒ | A description of all covariates tested |
| ☐ | ☒ | A description of any assumptions or corrections, such as tests of normality and adjustment for multiple comparisons |
| ☐ | ☒ | A full description of the statistical parameters including central tendency (e.g. means) or other basic estimates (e.g. regression coefficient) AND variation (e.g. standard deviation) or associated estimates of uncertainty (e.g. confidence intervals) |
| ☐ | ☒ | For null hypothesis testing, the test statistic (e.g. *F*, *t*, *r*) with confidence intervals, effect sizes, degrees of freedom and *P* value noted<br>*Give P values as exact values whenever suitable.* |
| ☒ | ☐ | For Bayesian analysis, information on the choice of priors and Markov chain Monte Carlo settings |
| ☒ | ☐ | For hierarchical and complex designs, identification of the appropriate level for tests and full reporting of outcomes |
| ☐ | ☒ | Estimates of effect sizes (e.g. Cohen's *d*, Pearson's *r*), indicating how they were calculated |

*Our web collection on statistics for biologists contains articles on many of the points above.*

## Software and code

Policy information about availability of computer code

| | |
|---|---|
| Data collection | The Cancer Genome Atlas(TCGA) GBM and GTEX normal tissue samples datasets were obtained from the UCSC Xena browser tool. RNA-seq data generated for this study have been deposited in the Gene Expression Omnibus (GEO) under accession number GSE291427. |
| Data analysis | The GraphPad Prism 10 software (RRID: SCR_002798).FastQC software (RRID:SCR_014583). HISAT2 (RRID: SCR_015530). SAMtools (RRID: SCR_002105). HTseq-count program (RRID: SCR_005514).Bioconductor's DEseq2 software (RRID:SCR_015687). |

For manuscripts utilizing custom algorithms or software that are central to the research but not yet described in published literature, software must be made available to editors and reviewers. We strongly encourage code deposition in a community repository (e.g. GitHub). See the Nature Portfolio guidelines for submitting code & software for further information.

## Data

Policy information about availability of data

All manuscripts must include a data availability statement. This statement should provide the following information, where applicable:

- Accession codes, unique identifiers, or web links for publicly available datasets
- A description of any restrictions on data availability
- For clinical datasets or third party data, please ensure that the statement adheres to our policy

RNA-seq data generated for this study have been deposited in the Gene Expression Omnibus (GEO) under accession number GSE291427. The dataset is currently private but accessible to reviewers via token: ijwtuwembvmzhob.

## Research involving human participants, their data, or biological material

Policy information about studies with human participants or human data. See also policy information about sex, gender (identity/presentation), and sexual orientation and race, ethnicity and racism.

| | |
|---|---|
| Reporting on sex and gender | NA |
| Reporting on race, ethnicity, or other socially relevant groupings | NA |
| Population characteristics | NA |
| Recruitment | NA |
| Ethics oversight | NA |

Note that full information on the approval of the study protocol must also be provided in the manuscript.

# Field-specific reporting

Please select the one below that is the best fit for your research. If you are not sure, read the appropriate sections before making your selection.

- [x] Life sciences
- [ ] Behavioural & social sciences
- [ ] Ecological, evolutionary & environmental sciences

For a reference copy of the document with all sections, see nature.com/documents/nr-reporting-summary-flat.pdf

# Life sciences study design

All studies must disclose on these points even when the disclosure is negative.

| | |
|---|---|
| Sample size | Sample size was based on standard experimental design in molecular biology. For most experiments (e.g. qPCR, ChIP, luciferase assays), at least three independent biological replicates were performed. Sample size was not determined by statistical power analysis but reflects accepted practices for reproducibility and detection of biologically relevant effects. |
| Data exclusions | No data were excluded from the analyses unless explicitly justified. |
| Replication | three independent experiments, each experiment with 2 to 5 technical replicates. |
| Randomization | Randomization was not applicable as experimental groups consisted of defined conditions (e.g., control vs. KANSL2 knockdown). Cell lines were assigned to groups based on treatment, not randomized. |
| Blinding | Experiments were not blinded. Quantification of immunofluorescence images was performed using automated thresholding and segmentation pipelines to reduce operator bias. |

# Reporting for specific materials, systems and methods

We require information from authors about some types of materials, experimental systems and methods used in many studies. Here, indicate whether each material, system or method listed is relevant to your study. If you are not sure if a list item applies to your research, read the appropriate section before selecting a response.

## Materials & experimental systems

| n/a | Involved in the study | |
|---|---|---|
| [ ] | [x] | Antibodies |
| [ ] | [x] | Eukaryotic cell lines |
| [x] | [ ] | Palaeontology and archaeology |
| [x] | [ ] | Animals and other organisms |
| [x] | [ ] | Clinical data |
| [x] | [ ] | Dual use research of concern |
| [x] | [ ] | Plants |

## Methods

| n/a | Involved in the study | |
|---|---|---|
| [x] | [ ] | ChIP-seq |
| [ ] | [x] | Flow cytometry |
| [x] | [ ] | MRI-based neuroimaging |

## Antibodies

| | |
|---|---|
| Antibodies used | KANSL2 HPA038497 RRID:AB_10674685 Sigma-Aldrich, KANSL1 STJ112653 RRID: AB_3665870 St John´s laboratories, KANSL3 STJ110533 RRID: AB_3665871 St John´s laboratories, KAT8/MOF Ab200660 RRID:AB_2891127 Abcam, FBL sc166000 RRID:AB_2105803 Santa Cruz<br>Biotechnology, UBF sc13125 RRID:AB_671403 Santa Cruz |

| | |
|---|---|
| | Biotechnology, PUROMYCIN MABE343 RRID:AB_2566826 Merck, Beta-Actin sc-47778 RRID:AB_626632 Santa Cruz<br>Biotechnology, RPS3 9538 RRID:AB_10622028 Cell signaling<br>technologies, RPS18 Ab91293 RRID:AB_2050267 Abcam, GAPDH ab8245 RRID:AB_2107448 Abcam, H4 ab10158<br>RRID:AB_296888<br>Abcam, H4K5ac 8647<br>RRID:AB_11217428<br>Cell signaling<br>technologies, H4K8ac ab45166<br>RRID:AB_732937<br>Abcam, H4K16ac 13534<br>RRID:AB_2687581<br>Cell signaling<br>technologies, Goat Anti-Mouse<br>IgG-HRP<br>1706516<br>RRID:AB_2921252<br>BIORAD, Goat Anti-Rabbit<br>IgG-HRP<br>1721019 RRID:AB_11125143<br>BIORAD, Goat anti-Mouse<br>IgG (H+L) Highly<br>Cross-Adsorbed<br>Secondary<br>Antibody, Alexa<br>Fluor™ Plus 488<br>A32723<br>RRID:AB_2633275<br>Thermo Fisher, Goat anti-Rabbit<br>IgG (H+L) Highly<br>Cross-Adsorbed<br>Secondary<br>Antibody, Alexa<br>Fluor™ Plus 555<br>A32732 RRID:AB_2633281 Thermo Fisher |
| Validation | Each antibody was checked for its specifity. |

## Eukaryotic cell lines

Policy information about cell lines and Sex and Gender in Research

| | |
|---|---|
| Cell line source(s) | G08 GBM IV derived cell line. NP WA09. F18-1 GBM grade 4, IDH-wildtype, MGMT promoter methylated. F2-4 grade 4 IDH-mutant astrocytoma. Lines U87, U251, HEK-293, and HeLa were acquired from ATCC either directly or from colleagues. |
| Authentication | Short Tandem Repeat (STR) profiling for cell lines from ATCC. |
| Mycoplasma contamination | Regularly checked for the absence of mycoplasma by PCR detection. |
| Commonly misidentified lines<br>(See ICLAC register) | NA |

## Plants

| | |
|---|---|
| Seed stocks | NA |
| Novel plant genotypes | NA |
| Authentication | NA |

# Flow Cytometry

## Plots

Confirm that:

- [x] The axis labels state the marker and fluorochrome used (e.g. CD4-FITC).
- [x] The axis scales are clearly visible. Include numbers along axes only for bottom left plot of group (a 'group' is an analysis of identical markers).
- [x] All plots are contour plots with outliers or pseudocolor plots.
- [x] A numerical value for number of cells or percentage (with statistics) is provided.

## Methodology

| | |
|---|---|
| Sample preparation | see materials and methods |
| Instrument | FACS Canto II instrument (BD Biosciences, USA) |
| Software | Floreada.io |
| Cell population abundance | Cell cycle distribution was assessed by PI staining in fixed cells. Relative abundance of cells in G0/G1, S, and G2/M was quantified using DNA content histograms. |
| Gating strategy | Cells were gated on FSC/SSC to exclude debris and doublets. PI fluorescence (linear scale) was used to quantify DNA content. G0/G1, S, and G2/M phases were defined based on DNA content histograms and quantified using Watson Pragmatic model in Floreada.io. |

- [x] Tick this box to confirm that a figure exemplifying the gating strategy is provided in the Supplementary Information.
